# Detection of SARS-CoV-2 Antibodies: Comparison of Enzyme Immunoassay, Surrogate Neutralization and Virus Neutralization Test

**DOI:** 10.3390/antib12020035

**Published:** 2023-05-10

**Authors:** Tatjana Vilibic-Cavlek, Maja Bogdanic, Ema Borko, Zeljka Hruskar, Denis Zilic, Thomas Ferenc, Irena Tabain, Ljubo Barbic, Mateja Vujica Ferenc, Ivana Ferencak, Vladimir Stevanovic

**Affiliations:** 1 Department of Virology, Croatian Institute of Public Health, 10000 Zagreb, Croatia; 2 School of Medicine, University of Zagreb, 10000 Zagreb, Croatia; 3 Axon Lab d.o.o., 10000 Zagreb, Croatia; 4 Clinical Department of Diagnostic and Interventional Radiology, Merkur University Hospital, 10000 Zagreb, Croatia; 5 Department of Microbiology and Infectious Diseases with Clinic, Faculty of Veterinary Medicine, University of Zagreb, 10000 Zagreb, Croatia; 6 Department of Obstetrics and Gynecology, University Hospital Center Zagreb, 10000 Zagreb, Croatia

**Keywords:** SARS-CoV-2, COVID-19, antibodies, EIA, surrogate neutralization test, virus neutralization test

## Abstract

Background: Since sensitivity and specificity vary widely between tests, SARS-CoV-2 serology results should be interpreted with caution. Methods: The study included serum samples from patients who had recovered from COVID-19 (*n* = 71), individuals vaccinated against SARS-CoV-2 (*n* = 84), and asymptomatic individuals (*n* = 33). All samples were tested for the presence of binding antibodies (enzyme immunoassay; EIA), neutralizing (NT) antibodies (virus neutralization test; VNT), and surrogate NT (sNT) antibodies (surrogate virus neutralization test; sVNT) of SARS-CoV-2. Results: SARS-CoV-2-binding antibodies were detected in 71 (100%) COVID-19 patients, 77 (91.6%) vaccinated individuals, and 4 (12.1%) control subjects. Among EIA-positive samples, VNT was positive (titer ≥ 8) in 100% of COVID-19 patients and 63 (75.0%) of the vaccinated individuals, while sVNT was positive (>30% inhibition) in 62 (87.3%) patients and 59 (70.2%) vaccinated individuals. The analysis of antibody levels showed a significant moderate positive correlation between EIA and VNT, a moderate positive correlation between EIA and sVNT, and a strong positive correlation between VNT and sVNT. The proportion of positive sVNT detection rate was associated with VNT titer. The lowest positivity (72.4%/70.8%) was detected in samples with low NT titers (8/16) and increased progressively from 88.2% in samples with titer 32 to 100% in samples with titer 256. Conclusions: sVNT appeared to be a reliable method for the assessment COVID-19 serology in patients with high antibody levels, while false-negative results were frequently observed in patients with low NT titers.

## 1. Introduction

Serological tests have the potential to significantly enhance the capacity to diagnose severe acute respiratory syndrome coronavirus 2 (SARS-CoV-2) in the current coronavirus disease (COVID-19) pandemic and have broad clinical applications, including in analyzing the immune response and identifying asymptomatic cases and those in the population who may be immune [1]. Numerous serological tests may be used to detect binding antibodies to SARS-CoV-2; however, they do not provide information on the potency of functional antibodies that may be linked to protective responses [2]. Since the sensitivity and specificity vary widely between tests, the serology results should be interpreted with caution.

Several serological tests for the diagnosis of SARS-CoV-2 are commercially available. These detect antibodies to assess the virus spike receptor binding domain (RBD), the whole-spike (S) antigen, the nucleocapsid (N) antigen, or all three [3]. Rapid immunochromatographic tests (ICT) and enzyme immunoassays (EIA) are widely in use as screening tests that qualitatively or semi-quantitatively measure the presence of IgG, IgM, and IgA antibodies [4]. However, due to the possible cross-reactions of binding antibodies with seasonal coronaviruses, a virus neutralization test (VNT) in cell culture remains the gold standard serological test for use in SARS-CoV-2 diagnosis. While a positive EIA result, even if specific, provides evidence of prior SARS-CoV-2 infection, it is not an assurance of protective immunity. Conversely, the presence of neutralizing (NT) antibodies correlates with protection [2,3].

VNTs are labor-intensive, difficult to standardize, and require the handling of live viruses in biosafety level 3 (BSL-3). Surrogate virus neutralization tests (sVNT) were developed in order to overcome these limitations of the VNT [4]. Several sVNTs are commercially available. The majority of these tests use the principle of an EIA to measure the NT capacity of anti-SARS-CoV-2 antibodies by inhibiting the interactions between the receptor-binding domain (RBD) of the S protein and angiotensin-converting enzyme 2 (ACE-2) cell receptors, mimicking the neutralization process. The percentage of binding inhibition between RBD and ACE-2 is then translated into percentage neutralization [5,6,7]. In contrast to the VNT, an sVNT can be performed in the process of BSL-2 containment. In addition, the sVNT has the advantages of technical simplicity and short duration (requiring only a few hours for completion) [8,9].

While in the natural SARS-CoV-2 infection, antibodies can be produced against all virus epitopes, the SARS-CoV-2 vaccine triggers the development of only S-protein-targeting antibodies [10].

Some studies compared different serological methods in the diagnosis of COVID-19. However, these studies have predominantly investigated the EIA and VNT, without many reports on the fluorescence immunoassay (FIA) in the detection of SARS-CoV-2 infection. This study aimed to evaluate the effectiveness of three serological tests for detecting SARS-CoV-2 antibodies in patients with COVID-19 and vaccinated individuals: EIA (binding antibodies), FIA (surrogate NT antibodies), and VNT (NT antibodies).

## 2. Materials and Methods

### 2.1. Samples

A total of 155 serum samples from patients who recovered from COVID-19 and had this confirmed by RT-PCR (*n* = 71) and individuals vaccinated against SARS-CoV-2 (*n* = 84) were included in the study. In addition, the study included samples from individuals who were asymptomatic at the time of testing and reported no recent febrile disease (control group, *n* = 33). All samples were tested for the presence of SARS-CoV-2-binding antibodies using EIA, in the presence of NT antibodies using VNT, and with surrogate NT (sNT) antibodies using FIA.

### 2.2. Enzyme-Linked Immunoassay

Initial serological testing (binding antibodies) was performed using an automated commercial EIA based on recombinant SARS-CoV-2 spike glycoprotein (S) and nucleocapsid protein (N) antigens (Vircell Microbiologists, Granada, Spain). The results were calculated, and the antibody index (AI) was expressed as: AI = (sample OD/cut-off serum mean OD) × 10. This was interpreted as follows: AI IgG < 4, negative, 4–6, borderline, >6, positive [11,12].

### 2.3. Fluorescence Immunoassay (Surrogate Neutralization Test)

Surrogate NT antibodies were detected using an automated commercial FIA (AFIAS COVID-19 nAb, Boditech Med Incorporated, Chuncheon-si, Gang-won-do, Republic of Korea). The test used a competitive immunodetection method for the qualitative determination of SARS-CoV-2 sNT antibodies that block the interaction between the RBD of the SARS-CoV-2 S glycoprotein with the ACE-2 cell surface receptor. The SARS-CoV-2 sNT present in the serum sample bound to the fluorescence-labeled SARS-CoV-2 RBD antigen and formed a complex. The complex migrated onto the nitrocellulose matrix with immobilized ACE-2 and interfered with the binding of sNT antibodies and fluorescence-labeled RBD. The results were calculated on the basis of inhibition rate (%) and interpreted as follows: cut-off index (COI; %) <30 negative; >30 positive.

### 2.4. Virus Neutralization Test

NT antibodies were detected using a VNT in cell culture. SARS-CoV-2, isolated in Vero E6 cells (ATCC CRL-1586) from a Croatian COVID-19 patient, was used as a stock virus. The virus titer (50% tissue culture infectious dose; TCID50) was calculated using the Reed and Muench formula. An equal volume (25 μL) of serial two-fold dilutions of heat-inactivated serum samples (30 min/56 °C) and 100 TCID50 of SARS-CoV-2 were mixed and incubated at 37 °C with CO_2_ for one hour. Finally, 50 μL of 2 × 10^5^ Vero E6 cells/mL were added to each well. To ensure optimal testing results, the virus antigen used in each run was back-titrated, and a positive sample with a known titer as well as a negative control sample were included in each plate. The plates were incubated at 37 °C with CO_2_ and, starting from the third day, the plates were checked for cytopathic effects. The titer was defined as the reciprocal of the highest serum dilution that showed at least 50% neutralization. NT antibody titer > 8 was considered positive [11,12].

## 3. Results

The overall SARS-CoV-2 positivity rates were 152 (80.0%) for binding antibodies (EIA), 134 (71.3%) for NT antibodies (VNT) and 123 (65.4%) for sNT antibodies (FIA, Table 1). SARS-CoV-2-binding antibodies (EIA) were detected in 71 (100%) COVID-19 patients and 77 (91.6%) vaccinated individuals. Among EIA-positive samples, NT antibodies (VNT) were detected in all (100%) COVID-19 patients and 63 (75.0%) vaccinated individuals, while sNT antibodies (FIA) were detected in 62 (87.3%) patients and 59 (70.2%) vaccinated individuals. In addition, 4 (12.1%) of samples from the control group were EIA positive, 2 (6.1%) were FIA positive, and no one was VNT positive.

**Figure 1:**
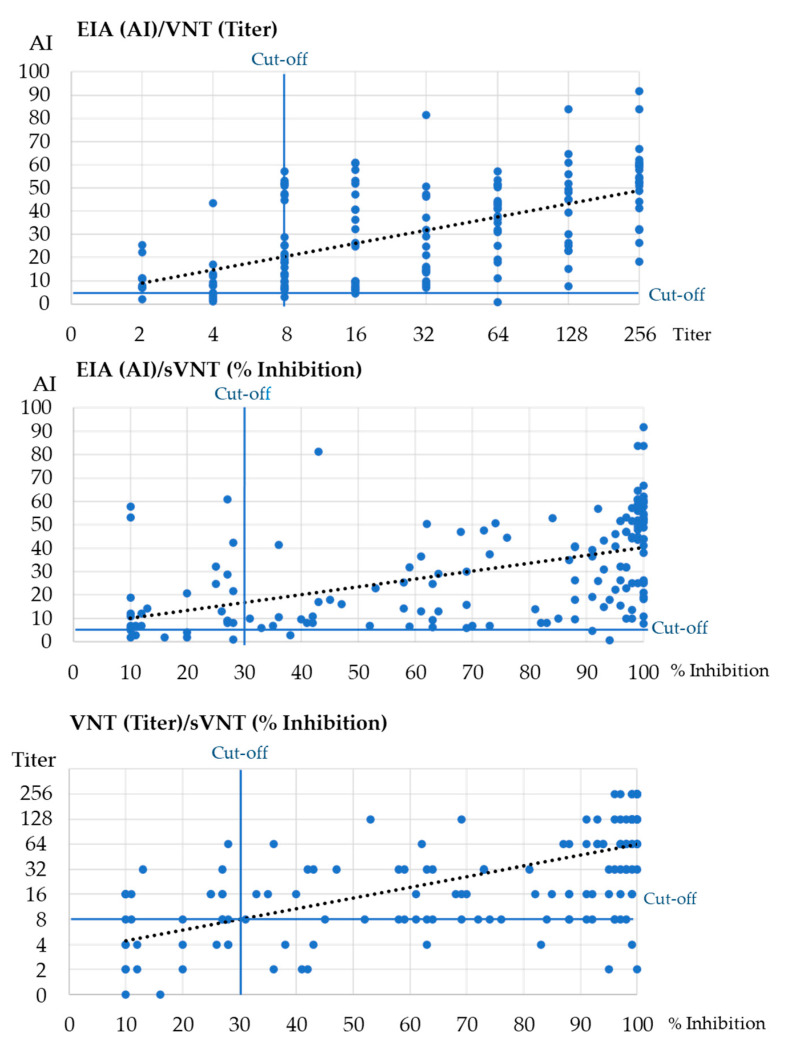
Comparison of EIA, FIA and VNT results in patients with COVID-19 and vaccinated individuals. The dotted line represents the trendline.

There was a significant moderate positive correlation between AI (EIA) and NT antibody titers (VNT) [Spearman’s rank rho = 0.572, *p* < 0.001], moderate positive correlation between AI (EIA) and sNT antibody titers (sVNT) [Spearman’s rank rho = 0.584, *p* < 0.001] and strong positive correlation between NT antibody titers (VNT) and sNT antibody titers (sVNT) [Spearman’s rank rho = 0.710, *p* < 0.001] (Figure 1).

**Figure 2:**
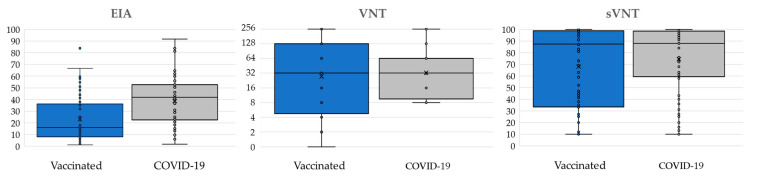
Antibody levels (median, IQR) detected by EIA (binding antibodies; AI), VNT (neutralizing antibodies; titer) and sVNT (binding inhibiting antibodies; % inhibition) according to vaccinal status; ◦ = inner and outlier points; x = mean values.

EIA, VNT, and sVNT antibody levels are presented in Figure 2. COVID-19 patients showed significantly higher binding antibody (EIA) levels (median AI = 41.5, IQR = 22.5–53.0) compared to vaccinated individuals (median AI = 16, IQR = 8.0–37.0; *p* < 0.001). However, there was no difference in the antibody level between groups for VNT (COVID-19 patients median NT antibody titer = 32, IQR = 4.3–128; vaccinated individuals median titer = 32, IQR = 8.3–64; *p* = 0.453) and sVNT (COVID-19 patients median binding antibodies: % inhibition = 88, IQR = 58–99; vaccinated individuals median % inhibition = 87.5; IQR = 33–99; *p* = 0.453).

A comparison of VNT- and sVNT-positive detection rates, determined according to NT antibody titers, is presented in Table 2. In a group of VNT-negative samples (titers 2 and 4), 9/21 (42.8%, 95%CI = 21.8–65.9) samples showed sNT antibodies. In a group of VNT-positive samples (titers > 8), the prevalence of sNT antibodies varied from 70.8 to 100%. The lowest prevalence rates (72.4 and 70.8%, respectively) were detected in samples with low NT antibody titers (8 and 16). In samples with NT antibody titers ≥ 32, the sVNT positivity increased progressively from 88.2% (samples with NT titer 32) to 100% (samples with NT titer 256).

The comparison of VNT and sVNT in VNT-positive COVID-19 patients and vaccinated individuals (NT antibody titer ≥ 8) showed no difference in the positive detection rate when using sVNT for all VNT titers (Table 3).

## 4. Discussion

Due to the cross-reactive nature of SARS-CoV-2 antibodies, serological methods have some limitations. These are especially important in children, who are more likely than adults to have had a recent infection with seasonal coronaviruses [13]. Cross-reactive false-positive results should be avoided, especially if seropositive individuals consider themselves to be immune to COVID-19. In these cases, the use of VNT or sVNT is needed to confirm the diagnosis of COVID-19.

This study compared three serological methods: EIA, VNT, and sVNT. The overall SARS-CoV-2 positivity rates were 80.0% for EIA, 71.3% for VNT, and 65.4% for sVNT. Among EIA-positive samples, 78.1% showed sNT antibodies, which was similar to the positivity rate obtained using sVNT reported in studies from Canada (75%) [14] and Utah (76.6%) [15].

Comparing antibody levels, our study showed a significant moderate positive correlation between EIA and VNT, a moderate positive correlation between EIA and sVNT, and a strong positive correlation between VNT and sVNT. Similarly, the agreement between sVNT values and VNT was found to be higher than the SARS-CoV-2–EIA agreement in an Italian study [2].

When analyzing the false positivity in the present study, the false-positive results were found in 12.1% of samples using EIA and 6.1% of samples using sVNT (FIA). Some other studies found an even higher non-NT (false-positive) antibody detection rate, generating positive results for 32.4% of the samples confirmed negative using a plaque reduction neutralization test (PRNT50). According to this observation, caution should be used in interpreting sVNT results since samples that tested positive using an sVNT may fail to neutralize SARS-CoV-2 in vitro as well as in vivo conditions [16].

In addition to the sensitivity and specificity of the serological methods used, SARS-CoV-2 antibody testing also obtains certain false positives due to endogenous and exogenous factors which may interfere with the results. The most common endogenous interferences include rheumatoid factors, heterophile antibodies, complement, and cross-antigens. Exogenous interference occurs mainly via incomplete coagulation or from the contamination of the sample [17]. Patients with connective tissue diseases can have a high level of ACE-2 antibodies that can cause false-positive sVNT [18]. It has been documented that the false-positive test (EIA and sVNT) can be the result of prior infection with seasonal coronaviruses [19]. The study conducted on samples collected before the onset of the COVID-19 pandemic reported the occurrence of cross-reaction to acute infections with different pathogens, namely *Rickettsia typhi*, *Salmonella typhi*, *Leptospira* spp., and influenza B virus [20]. Some studies have reported that the hemagglutinin of the influenza B virus cross-reacts with anti-SARS-CoV-2 non-NT antibodies [21].

Positive detection rates in our study were associated with VNT titers. The highest positivity detection (100%) was found in samples with the highest NT antibody titer (256). The lowest positivity rates were observed in samples with NT antibody titer of 8 and 16 (72.4 and 70.8%, respectively), while they increased progressively from 88.2% in samples with a VNT titer of 32 to 100% in samples with a titer of 256.

A study from Germany showed that binding inhibition values (sVNT) were significantly higher in vaccinees (median 95.7, IQR = 88.1–96.8) compared to convalescent COVID-19 patients (median 52.9, IQR = 31.2–76.2) [10]. In contrast, our results showed no significant difference between the binding antibody levels (% inhibition) detected by sVNT in COVID-19 patients (median 88, IQR = 58–99) and vaccinated individuals (median 87.5, IQR = 33–99). In addition, while PRNT50 titers were also found to be higher in vaccinated individuals compared to convalescent COVID-19 patients in a German study (median 119.8, IQR = 56.7–169 vs. median 49.1, IQR = 20–62), similar results were not observed in our study (VNT titer median 32, IQR = 4.3–128 vs. median 32, IQR = 8.3–64). However, it is important to note the different sampling times in German patients. Samples from vaccinees were taken later (median 10 weeks after the second dose) compared to samples from COVID-19 patients (median 5 weeks after the symptom onset). This could at least partly influence the serology results. Some studies indicated that SARS-CoV-2 NT antibodies decline weeks after infection. Therefore, the sampling time is an important factor that should be used into account for determining protective immunity [16,22,23].

In the present study, sNT antibodies had false-negative results in 21.9% of samples. False-negative rates depended on the VNT titers. The highest negativity rates were in samples with low VNT titers (54.6% in samples with NT titer 8 and 50.5% in samples with NT titer 16). Like in our study, PRNT-positive samples were falsely identified as negatively utilizing sVNT in a substantial proportion of the cases (23.6%) in one German study [10]. In another German study, the proportion of false-negative samples detected using two sVNT was lower in convalescent serum samples (15.6 and 6.7%), while in post-vaccinal samples no false-negative results were found [24].

It has been demonstrated that SARS-CoV-2 infection results in the development of antibodies against the RBD and the S1 domain, but also against the S2 as well as N domain [25]. While antibodies against the N protein are likely to be non-neutralizing, antibodies against the N-terminal domain of S1 (outside of the RBD) have demonstrated the potential to be neutralizing [26]. Additionally, antibodies that target the SARS-CoV S2 domain exhibit neutralizing properties [27]. False-negative sVNT results might be attributed to the fact that the VNT is able to detect neutralization, irrespective of specific epitopes. In contrast, sVNT is only able to identify antibodies that function by blocking the interaction between the ACE-2 receptor and the viral RBD, which is unquestionably a general limitation of EIA-based methods [9].

In general, some similar studies were found, like in this study, the higher the VNT titer, the less likely false negative sNT results were obtained. A study conducted in Switzerland among patients with COVID-19 showed an overall clinical sensitivity of 80.3%. Comparing an sVNT with VNT, the analytical sensitivity was determined to be 74.3% (56.4–86.9%) and 98.2% (89.4–99.9%), respectively, for samples with NT titers ≥10 to <40 and ≥40 to <160. Only samples with VNT titer ≥160 always showed blocking activity in the sVNT [4]. Additionally, it was demonstrated that individuals who develop S-specific binding antibodies were also likely to produce neutralizing IgG. However, the correlation between neutralization and N-specific IgG was consistently lower than the correlation between neutralization and S- and RBD-specific IgG [7].

The present study has some limitations that need to be addressed. For the majority of participants, data on the sampling time after COVID-19 infection and vaccination was not available, an issue which may interfere with the serology results. In addition, the study included a small number of samples. Both limitations should be taken into account when interpreting the results.

## 5. Conclusions

The NT antibody titer is an important indicator of immunity to SARS-CoV-2. Neutralization tests are powerful diagnostic methods for COVID-19 diagnosis and for use in the evaluation of post-vaccinal responses. In comparison with VNT, sVNT does not have safety issues since it does not require exposure to the live virus and is faster, easier to perform, and has a lower cost than traditional VNT. However, sVNT cannot detect all neutralizing antibodies, only antibodies to RBD. Due to the high SARS-CoV-2 mutation rate, the circulating strains are constantly evolving and changing in terms of their antigenicity. Therefore, it is expected that immune responses will occur in the population due to natural infection and vaccination with newly updated boosters. The connection between RBD and ACE-2, where NT antibodies bind most frequently, is affected by antigenicity modification. Thus, the use of sVNT can bias the detection of NT antibodies. For these reasons, many countries prefer VNT as a gold standard serological method. sVNT appeared to be a reliable method for COVID-19 serology in patients with high antibody levels; however, it cannot replace traditional VNTs since false-negative results are frequently observed in patients with low NT antibody levels.

## Figures and Tables

**Table 1:** SARS-CoV-2 detection rates using EIA, VNT and sVNT.

Group	EIA		VNT		sVNT (FIA)	
	N (%)	95%CI	N (%)	95%CI	N (%)	95%CI
COVID-19 patients; *n* = 71	71 (100)	94.0–100 ^1^	71 (100)	94.0–100 ^1^	62 (87.3)	77.3–94.0
Vaccinated individuals; *n* = 84	77 (91.6)	83.5–96.5	63 (75.0)	64.3–83.8	59 (70.2)	59.3–79.7
Control group; *n* = 33	4 (12.1)	3.4–28.2	0 (0)	0–10.5 ^1^	2 (6.1)	0.7–20.2
Total; *n* = 188	152 (80.0)	74.5–86.2	134 (71.3)	64.2–77.6	123 (65.4)	58.2–72.2

^1^ One-sided 97.5% confidence interval.

**Table 2:** Comparison of VNT and sVNT detection rates in COVID-19 patients and vaccinated individuals (*n* = 153).

VNT Result	VNT Titer	VNT N (%)	sVNT N (%) Positive	95%CI
VNT negative (*n* = 21)	2	9 (5.8)	4 (44.4)	13.7–78.8
	4	12 (7.8)	5 (41.7)	15.2–72.3
VNT positive (*n* = 132)	8	29 (18.9)	21 (72.4)	52.8–87.2
	16	24 (15.7)	17 (70.8)	58.9–87.3
	32	17 (11.1)	15 (88.2)	63.6–98.5
	64	20 (13.1)	18 (90.0)	68.3–98.7
	128	17 (11.1)	16 (94.1)	71.3–99.8
	256	25 (16.3)	25 (100)	86.3–100 *

VNT = virus neutralization test; sVNT = surrogate virus neutralization test; * one-sided 97.5% confidence interval.

**Table 3:** SARS-CoV-2-positive detection rate according to vaccinal status in VNT-positive participants.

VNT Titer	COVID-19 Patients (*n* = 71)			Vaccinated Individuals (*n* = 50)			*p*
	VNT Positive	sVNT Positive		VNT Positive	sVNT Positive		
	N	N (%)	95%CI	N	N (%)	95%CI	
8	18	14 (77.8)	52.4–93.6	11	5 (45.4)	16.7–76.6	0.755
16	14	10 (71.4)	41.9–91.6	10	5 (50.0)	18.7–81.3	0.285
32	10	9 (90.0)	55.5–99.7	7	6 (85.7)	42.1–99.6	0.787
64	13	13 (100)	75.3–100 *	7	6 (85.7)	42.1–99.6	0.162
128	7	7 (100)	59.0–100 *	10	10 (100)	69.1–100 *	1.000
256	9	9 (100)	66.3–100 *	18	18 (100)	81.5–100 *	1.000

* One-sided 97.5% confidence interval.

## Data Availability

All related data and methods are presented in this paper. Additional inquiries should be addressed to the corresponding author.
